# Tsrp1 Is a Novel Cyclic Diguanosine Monophosphate Effector that Plays a Role in the Organic-Solvent Tolerance of *Rhodococcus ruber*

**DOI:** 10.4014/jmb.2410.10039

**Published:** 2025-03-26

**Authors:** Fan Zhang, Han Yu, Lei Ai, Yixin Hao, Ren Peng

**Affiliations:** College of Life Science, Jiangxi Normal University, Nanchang 330022, P.R. China

**Keywords:** Cyclic diguanosine monophosphate, effector, organic-solvent tolerance, transcriptomic analysis

## Abstract

Cyclic diguanosine monophosphate is a ubiquitous second messenger that regulates diverse cellular processes. *Rhodococcus ruber* SD3 has potential for use in removing environmental pollutants such as phenol and toluene. In this study, Tsrp1 was found to be a novel cyclic diguanosine monophosphate effector in this strain. The interaction between Tsrp1 and c-di-GMP was verified by surface plasmon resonance, and the dissociation constant was 64 ± 6.84 μM. Moreover, in comparison with the wild-type strain, the recombinant *R. ruber* SD3 strain, which exhibited elevated levels *tsrp1* gene expression, demonstrated enhanced growth in the presence of toluene and phenol. Both recombinant *R. ruber* SD3 and the wild-type strain completely degraded toluene (0.3 g/l, 0.6 g/l and 0.9 g/l) and phenol (0.6 g/l, 0.8 g/l and 1.0 g/l) in 72 h. Furthermore, differential expression of key genes encoding transcription factors was identified based on the transcriptomic comparison between the two strains. This study is the first to describe a novel cyclic diguanosine monophosphate effector and its role in the characteristics of *R. ruber* SD3, which will shed new light on the mechanisms underlying the organic solvent tolerance of *R. ruber* SD3.

## Introduction

Organic solvents include various organic compounds, such as toluene, acetone, n-butanol and phenol, which can solubilize chemical substances. Modern industry is characterized by the wide application of numerous organic solvents. They are used as materials or intermediates for improving the solubility of substrates. Nevertheless, if organic solvent waste is discharged without treatment in the process of manufacturing, it can cause serious harm to the environment and severely threaten human health [1]. For example, toluene, a versatile organic solvent, is industrially exploited for the production of polyols, antioxidants, corrosion inhibitors, polyurethane, adhesives, and polyethylene terephthalate (PET) [2]. However, toluene is detrimental to human health, even at low concentrations. It harms the liver, lung, and nervous system and results in malignant tumors owing to its high toxicity and mutagenicity [3, 4]. To degrade toluene, photocatalysis, chemical catalysis, and biotransformation have been utilized. Liu *et al*. demonstrated nanodiamond-decorated ZnO catalysts for the photocatalytic degradation of toluene [5]. In the Mn_2_O_3_/γ-Al_2_O_3_+double dielectric barrier discharge system, a toluene degradation efficiency of 93.3% was achieved, with a specific energy input of 700 J/L [6]. Using Fe(III)NTA and nitrate as electron acceptors, Weelink *et al*. reported that *Georgfuchsia toluolica* G5G6 was able to degrade toluene [7]. Phenol is another kind of mono-substituted aromatic hydrocarbon with a special odor and high reactivity. It is used for the manufacture of plastic, pharmaceuticals, paints, adhesives and disinfectants [8]. Nevertheless, phenol is easily absorbed through the respiratory tract and skin. Short-term exposure to phenol results in hemolytic anemia, poisoning, and central nervous system damage [9]. Thus, phenol is considered one of 126 priority pollutants by the U.S. Environmental Protection Agency [10]. Currently, various methods are used to remove phenol from the surrounding environment. Bahrudin reported that an immobilized TiO_2_/AC bilayer photocatalyst was successfully fabricated for phenol degradation in aqueous media [11]. *Isochrysis galbana* Parke MACC/H59 was subjected to adaptive laboratory evolution to obtain two strains (PAS-1 and PAS-2) with greater phenol degradation than the original alga [12].

Among the numerous methods for tackling organic solvents, bioremediation is considered promising owing to its low cost and nonsecondary pollution [13]. The process of bioremediation involves leveraging the metabolic capabilities to convert or break down harmful pollutants into less dangerous compounds, thereby incorporating them into the surrounding biogeochemical cycles. Organic-solvent-tolerant microorganisms can thrive in the presence of organic solvents and can usually degrade organic solvents. Hence, they exhibit potential in the field of bioremediation. *Rhodococcus* is a group of obligately aerobic, gram-positive bacteria that are widely distributed. *Rhodococcus* species possess a diverse array of catabolic functions, distinctive enzymatic profiles, and the capacity to synthesize biosurfactants. Additionally, they lack catabolic repression, enabling them to decompose an extensive variety of contaminants, even when simple nutrients are readily available [14]. Thus, *Rhodococcus* strains find wide application for the bioremediation of different xenobiotics, such as toluene [15], phenol [16], propene [17], fluoranthene [18], and dibenzothiophene [19]. For instance, *Rhodococcus* sp. EH831 could aerobically degrade benzene, tolulene, ethylbenzene, xylene, and methyl tert-butyl ether. The specific degradation rates of these five compounds were 234, 913, 131, 184, and 139 μmol g(DCW)^−1^ h^−1^, respectively [15].

*R. ruber* SD3 is an organic-solvent-tolerant strain that we isolated from soil. We used a quantitative proteomics approach to investigate protein expression changes in *R. ruber* SD3 upon toluene stress. There was an uncharacteristic protein (A0A098BH47) with the highest upregulation (Log_2_FC) of 3.34 [20]. The present paper is intended to reveal the function of this protein (Tsrp1) via the evidence of interaction between Tsrp1 and c-di-GMP, and by comparing the phenotype and transcriptome of Tsrp1-overexpressing strain and wild-type *R. ruber* SD3.

## Materials and Methods

### Bacterial Strains and Plasmids

*R. ruber* SD3 was screened and isolated from polluted sludge and deposited in the China Center for Type Culture Collection (M2012035). This strain can degrade some organic solvents, such as phenol and toluene [20]. The pET-21b and pET-28a plasmids were used for the recombinant expression of Tsrp1 in *E. coli* BL21(DE3). The pNV18 plasmid was used to increase the expression of Tsrp1 in *R. ruber* SD3.

### Expression and Purification of Recombinant Tsrp1

The sequence of the *tsrp1* gene was determined using the T/A cloning method before being submitted to GenBank under accession no. MK371001 [21]. The *tsrp1* gene with restriction endonuclease (NdeI and EcoRI) sites was synthesized by General Bio Co., Ltd. (China) based on sequence codon optimization. The gene and pET-21b were digested with NdeI and EcoRI. Enzymatic digestion products were checked by 1% agarose gel electrophoresis and recovered by an Agarose Gel DNA Recovery Kit (Omega Bio-tek, USA). The recovered gene was ligated to the digested pET-21b to form the recombinant plasmid pET-21b-*tsrp1*, which was then transformed into *E. coli* Top 10. The positive clones were incubated in LB broth medium for 24 h. The recombinant plasmid was extracted with a Mini-Prep Plasmid Extraction Kit (Solarbio, China) and then transformed into *E. coli* BL21 (DE3) to obtain a recombinant *E. coli* strain harboring pET- 21b-*tsrp1*. Similarly, the *tsrp1* gene with restriction endonuclease (BamHI and EcoRI) sites was codon-optimized and then synthesized. A recombinant *E. coli* strain harboring pET-28a-*tsrp1* was constructed. These two recombinant *E. coli* BL21(DE3) strains were inoculated in LB broth supplemented with 100 μg/ml ampicillin at 37°C for 24 h. The culture was subsequently grown in fresh LB media at 37°C until the OD_A600_ reached 0.8. The expression of Tsrp1 was induced by adding IPTG (0.5 mM or 1.0 mM), followed by incubation at 37°C for 9 h. The cells were harvested by centrifugation at 5,000 ×*g* for 15 min, and then kept at -40°C for 30 min. Afterwards, a nondenatured lysing solution (50 mM NaH_2_PO_4_·H_2_O, 300 mM NaCl, pH 8.0) was mixed with the cells. The cells were lysed by ultrasonic waves (with an ultrasonic time of 3 s, a gap time of 5 s, the ultrasonic power of 60 W, and a total ultrasonic time of about 10 min). The sample was subjected to centrifugation at 12,000 ×*g* for 30 min. The total protein, supernatant, and precipitate were then subjected to SDS analysis to detect the expression of recombinant Tsrp1.

Next, His-tagged purification resin was pre-equilibrated with nondenatured lysing solution. The supernatant was then mixed with His-tagged purification resin at 4°C for 60 min to ensure the binding of recombinant Tsrp1 to the resin. The recombinant Tsrp1-bound resin was then loaded onto a column. Unbound protein was removed with a nondenatured lysing solution (containing 2 mM imidazole). Following that, the recombinant Tsrp1 was eluted with a nondenatured lysing solution (containing 50 mM imidazole). The samples from each purification step were then withdrawn for SDS-PAGE analysis. Finally, the purified recombinant Tsrp1 band was cut from gels, and the sequence of recombinant Tsrp1 was confirmed using liquid chromatography-tandem mass spectrometry (LC-MS/MS).

### Assay of c-di-GMP Binding to Tsrp1 by Localized Surface Plasmon Resonance

The binding of c-di-GMP to Tsrp1 was performed using localized surface plasmon resonance (LSPR). To measure the interaction between c-di-GMP and Tsrp1, Tsrp1 was immobilized onto the NTA chips. C-di-GMP was diluted in HEPES-buffered saline (pH 7.4) to concentrations of 500, 250, 125, or 62.5 μM and injected at 20 μl/min. The binding and disassociation times between recombinant Tsrp1 and c-di-GMP were 240 s and 480 s, respectively. The results were analyzed using the software TraceDrawer and the One-to-One model. BSA was used as a negative control.

Construction and Demonstration of Recombinant *R. ruber* SD3 Increasing the Expression of the *tsrp1* Gene The genome of *R. ruber* SD3 was extracted according to the method described by Yuan *et al*. [22]. We amplified the *tsrp1* gene by PCR in a thermal cycler using the following program: 94°C for 5 min; followed by 30 cycles of 94°C for 30 s, 60°C for 30 s, 72°C for 90 s, and a final extension of 10 min at 72°C. The forward primer (5’-CGGGATCCATGACCACCGCCAAGACC-3’) and reverse primer (5’-CCCAAGCTTCTTGAGGAGATCGCGGG-3’) sequences contained restriction endonuclease sites (BamHI and HindIII), respectively. PCR products were verified by 1% agarose gel electrophoresis and purified using the Gel Extraction Kit D2500 (Omega Bio-tek). Tsrp1 and pNV18 were digested by BamHI and HindIII, respectively. The digested fragments were also verified by agarose gel electrophoresis and recovered using the Gel Extraction Kit D2500 (Omega Bio-tek). A recombinant plasmid of cep1-pNV18 was constructed by ligating the purified fragments at 16°C overnight, and this plasmid was transformed into *E. coli* JM109. After growing recombinant *E. coli* JM109 overnight, cep1-pNV18 was extracted and electrotransformed into *R. ruber* SD3. The presence of recombinant *R. ruber* SD3 strain was verified by colony PCR. Quantitative real-time polymerase chain reaction was used to assess the difference in the expression of *tsrp1* between recombinant *R. ruber* SD3 and wild-type *R. ruber* SD3 in the presence of toluene and phenol, respectively. The primers used for qPCR are listed in Table S1.

### Growth Curves and Microscopic Observation of Recombinant *R. ruber* SD3 and Wild-Type *R. ruber* SD3

Recombinant *R. ruber* SD3 and wild-type *R. ruber* SD3 were grown in LB broth at 35°C and 200 rpm overnight. Five hundred microliters of the seed culture was transferred to 50 ml of LB broth. The cultivation was performed at 35°C and 200 rpm for 46 h. Cultures were withdrawn every two hours to measure the OD at 600 nm.

Cells were harvested by centrifugation at 4°C and 10,000 ×*g* and washed with 200 mM PBS (pH 7.4) three times. Then, the cells were fixed overnight at 4°C with 1 ml of glutaraldehyde solution (2.5%). After fixation, the glutaraldehyde solution was removed by centrifugation, and the cells were washed with PBS three times and subjected to dehydration using 1 ml of methanol for 20 min. The specimen was spotted on a silicon slide for drying and placed on a holder, which was then gilded for observation under a scanning electron microscope.

### Effects of Different Organic Solvents on the Growth of Recombinant *R. ruber* SD3 and Wild-Type *R. ruber* SD3

The seed culture was prepared as described above, and 1 ml of the seed culture was transferred to 50 ml of LB broth. Then, 0.02% (v/v) toluene and 0.08% (m/v) phenol were separately added to LB broth to study the effects of organic solvents on the growth of recombinant *R. ruber* SD3 and wild-type *R. ruber* SD3. The cultivation was performed at 35°C and 200 rpm for 72 h. Cultures were withdrawn to measure the OD at 600 nm every 12 h.

Cultures of recombinant *R. ruber* SD3 and wild-type *R. ruber* SD3 were spot-inoculated on LB solid plates containing phenol (0.08% m/V) according to standard agar dilution. Then, subsequent incubation was performed at 35°C for 12 h. To investigate the effects of toluene, the LB plate was overlaid with 300 μl of toluene (12.5% v/v), followed by the inoculation of the two strains at 35°C.

### Degradation of Toluene by Recombinant *R. ruber* SD3 and Wild-Type *R. ruber* SD3 and Assay of Toluene Content by GC

The seed culture was prepared as described above, and 2 ml of the seed culture was transferred to 3 ml of LB broth. Then, toluene at different concentrations (0.3 g/l, 0.6 g/l, and 0.9 g/l) was added to the LB broth. Cultures were incubated at 35°C and 200 rpm for 72 h and withdrawn to measure the residual toluene every 24 h. Briefly, cultures were centrifuged at 4°C and 10,000 r/min. The supernatant was transferred to a glass centrifuge tube and adjusted to pH 2.0 with 3 mol/l hydrochloric acid. Then, 2 g of ammonium sulphate and 5 ml of n-hexane were added, and the mixture was ultrasonicated for 30 min and centrifuged at 3,000 r/min for 5 min. The n-hexane fraction was removed from the mixture, after which the toluene extraction procedure was carried out once more. The n-hexane fraction was combined, dried over anhydrous sodium sulfate, and finally subjected to gas chromatography-mass spectrometry (GC-MS) analysis.

GC-MS analysis was performed on a Trace1300ISQ gas chromatograph and mass spectrometer with an HP-5ms column, which was run with an injection volume of 1 μl, a flow rate of 1.0 ml/min, and a running time of 2.6 min. The initial column temperature was 50°C; the temperature was maintained for 3 min, and the column was subsequently heated to 200°C at a rate of 20°C/min. The inlet temperature was 150°C, the detector temperature was 250°C, and the diversion ratio was 20:1.

### Degradation of Phenol by Recombinant *R. ruber* SD3 and Wild-Type *R. ruber* SD3 and Assay of Phenol Content by HPLC

The seed culture was prepared as described above, and 2 ml of the seed culture was transferred to 100 ml of LB broth. Then, phenol at different concentrations (0.6 g/l, 0.8 g/l, and 1.0 g/l) was separately added to the LB broth. Cultures were incubated for 72 h and withdrawn to measure the residual phenol. Briefly, cultures were centrifuged at 4°C and 10,000 r/min. The supernatant was subjected to filtration with a 0.22 μm membrane. HPLC was performed on a Shimadzu LC-2030C (Shimadzu Corp., Japan) with a ZORBAX Eclipse XDB-C18 column (Agilent, USA), which was run using methanol and water (80:20, v/v) as the mobile phase at a flow rate of 1.0 ml/min. Phenol absorbance was monitored at 270 nm using a UV detector.

### Transcriptome Analysis of Recombinant *R. ruber* SD3 and Wild-Type *R. ruber* SD3 by RNA-seq

Two percent seed culture of recombinant *R. ruber* SD3 and wild-type *R. ruber* SD3 was inoculated into 100 ml of LB broth medium containing 0.08% (m/v) phenol respectively. The culture was grown at 35°C and 180 rpm for 24 h. The cells were harvested by centrifugation at 4°C. Total RNA from cells was isolated with TRIzol Reagent. The concentration and purity of the extracted RNA were detected using a NanoDrop 2000 (Thermo Fisher Scientific, USA). Its integrity was confirmed by agarose gel electrophoresis. The RNA integrity was assayed using an Agilent 2100 system (Agilent, USA). The cDNA library was constructed using the TruSeq Stranded Total RNA Library Prep Kit (Illumina, USA). To contain A/U/C/G in the second chain of cDNA, dUTP was used for the synthesis of the second chain of cDNA in place of dTTP. The second chain of cDNA was digested prior to PCR amplification, whereby only the first chain of cDNA was included in the library. Sequencing was performed on an Illumina HiSeq 2500/Miseq platform (USA). A single run of Illumina sequencing produces billions of reads, and the quality of each read cannot be individually revealed for such a large amount of data. Thus, several statistical methods were used for the statistical analysis and quality control of the sequences. The mapped data (reads) were obtained by aligning the clean data (reads) after quality control with the reference genome. The data of raw sequence reads were deposited at NCBI (Project no. PRJNA1200282).

The functional unigenes were annotated according to six databases (NCBI nonredundant protein/nucleotide sequences, Protein family, Clusters of Orthologous Groups of proteins, Swiss-Prot, Kyoto Encyclopedia of Genes and Genomes, and Gene Ontology). Differential expression between recombinant *R. ruber* SD3 and wild-type *R. ruber* SD3 was analyzed using DESeq2 software to obtain differentially expressed genes (DEGs). The *p*-value was adjusted to control the false discovery rate. DEGs were identified as genes with *p*-adjust < 0.05 and |log_2_FC| ≥ 1. GO enrichment analysis of DEGs was performed using Goatools software. GO functions was considered significantly enriched when the adjusted *p*-value was <0.05. KEGG enrichment analysis of DEGs was performed using an R script. KEGG pathway function was recognized as significantly enriched when the adjusted *p*-value was <0.05. A subset of several transcriptional regulator genes was selected for validation of RNA-Seq by qRT-PCR. The sequences of all primers are given in Table S1.

### Quantitative Real-Time Polymerase Chain Reactions

Quantitative real-time polymerase chain reactions were carried out according to the procedure of Kuang *et al*.(2018). All the data were normalized to the 16S rRNA gene according to the 2^-ΔΔCT^ method [23].

## Results and Discussion

### Recombinant Expression of *tsrp1* in *E. coli* BL21(DE3)

**Fig. 1:**
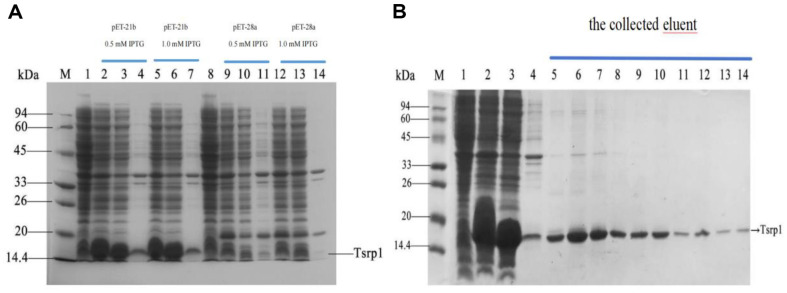
SDS-PAGE pattern of various samples prepared from different recombinant strains induced by IPTG at different concentrations and the purification process of Tsrp1 recombinant protein. (**A**) Marker (lane M); The bacterial extract obtained by disruption of the recombinant strain containing pET-21b-*tsrp1* plasmid or pET-28a-*tsrp1* plasmid not induced by IPTG (lane l and lane 8); The bacterial extract, the supernatant and the precipitate obtained by the disruption of the recombinant strain containing pET-21b-*tsrp1* plasmid induced by 0.5 mM IPTG (lanes 2-4); The bacterial extract, the supernatant and the precipitate obtained by the disruption of the recombinant strain containing pET-21b-*tsrp1* plasmid induced by 1.0 mM IPTG (lanes 5-7); The bacterial extract, the supernatant and the precipitate obtained by the disruption of the recombinant strain containing pET-28a-*tsrp1* plasmid induced by 0.5 mM IPTG (lane 9-11); The bacterial extract, the supernatant and the precipitate obtained by the disruption of the recombinant strain containing pET-28a-*tsrp1* plasmid induced by 1.0 mmol/l IPTG (lanes 12-14). (**B**) Marker (lane M); The bacterial extract obtained by disruption of the recombinant strain containing the pET-21b-*tsrp1* plasmid not induced by IPTG (lane 1); The bacterial extract (lane 2), the supernatant (lane 3) and the precipitate (lane 4) obtained by disruption of the recombinant strain containing the pET-21b-*tsrp1* plasmid induced by 0.5 mM IPTG; the collected eluent (lanes 5-14).

In a previous paper of ours, to study the organic solvent mechanism of *R. ruber* SD3, we compared the proteome of *R. ruber* SD3 in the presence of toluene stress with that of *R. ruber* SD3 devoid of toluene stress. Among all proteins, Tsrp1 exhibited the greatest upregulation, despite it being just an uncharacterized protein [20]. Interestingly, in another experiment that aimed to screen c-di-GMP effector proteins in *R. ruber* SD3 by the pulldown method, Tsrp1 also appeared among c-di-GMP effector protein candidates [22]. To confirm the interaction between Tsrp1 and c-di-GMP via localized surface plasmon resonance, an adequate quantity of Tsrp1 was obtained for the assay. To increase the expression of *tsrp1* in *E. coli*, codon optimization was first conducted. Moreover, to investigate the effects of different plasmids on protein expression, we constructed two recombinant *E. coli* strains harboring pET-21b-*tsrp1* and pET-28a-*tsrp1*. As shown in Fig. 1A, the expression of *tsrp1* with pET-21b (lane 2) was greater than the expression of *tsrp1* with pET-28a (lane 9), when IPTG (0.5 mM) was used for induction. A similar result was obtained in the presence of IPTG (1.0 mM). Thus, pET-21b was superior to pET-28a for the expression of tsrp1. For the recombinant *E. coli* strains harboring pET-21b-*tsrp1*, the expression of *tsrp1* induced by IPTG (0.5 mM) (lane 2) was slightly greater than that induced by IPTG (1.0 mM) (lane 5). Additionally, more recombinant Tsrp1 was detected in the supernatant of the cell lysate (lane 3) than in the precipitate of the cell lysate (lane 4). These results suggested that *tsrp1* was mainly expressed in its water-soluble form. Thus, we used the supernatant of the cell lysate for protein purification. Metal chelation affinity chromatography was utilized for the purification of recombinant Tsrp1 owing to its His-tag. Fig.1B confirmed the successful purification of recombinant Tsrp1 (lanes 5-14). LC-MS/MS was also used to verify the accurate expression and purification of recombinant Tsrp1. The coverage of the protein sequence with a confidence ≥ 95%was 99.28% (Fig. S1). The results demonstrated that the purified protein was our target protein.

### The Interaction Between Tsrp1 and c-di-GMP

**Fig. 2:**
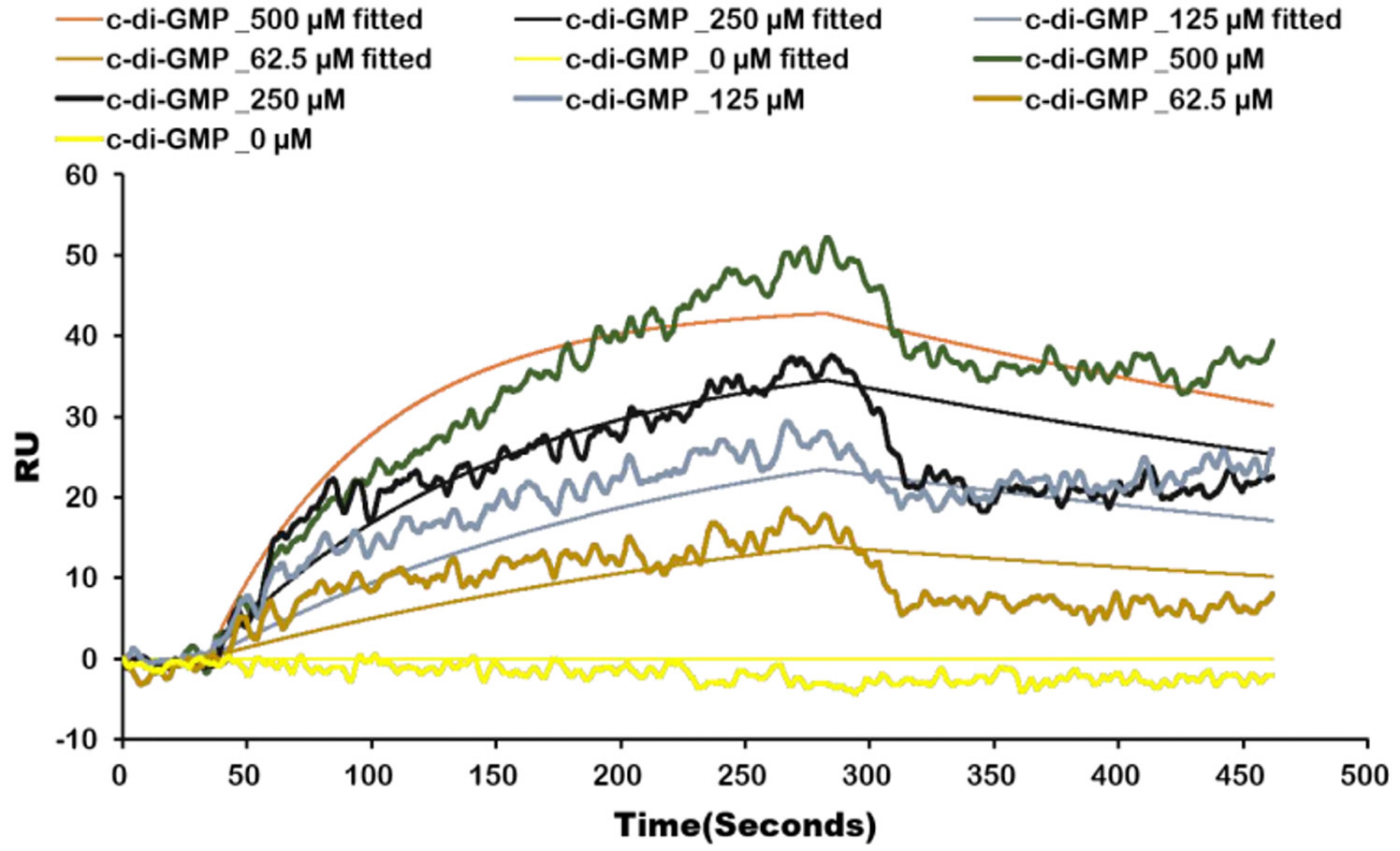
The binding curve of Tsrp1 and c-di-GMP by local surface plasmon resonance.

As a powerful technique for demonstrating interactions between biomolecules, LSPR is employed to study the kinetics of molecular binding by observing the surface plasmon resonance response in the time domain with high sensitivity [24]. Therefore, we also used this method to confirm the interaction between c-di-GMP and Tsrp1. As shown in Fig. 2, c-di-GMP had affinity for Tsrp1, with a Kd value of 64 ± 6.84 mM. Docking between c-di-GMP and Tsrp1 indicated that c-di-GMP could form stable conventional hydrogen bonds with Thr 15 and Thr 45, carbon hydrogen bonds with Asp 18, Ala 44, and Gln 99, pi-alkyl interactions with Val 12, Ala 41, Ala 44, Ala 48, Ala 51, Val 92, and Leu 96, and pi-pistacked interactions with Tyr 88 [21]. In this regard, Tsrp1 was described as a c-di-GMP effector according to the results of LSPR and docking.

In bacteria, c-di-GMP, a versatile and ubiquitous second messenger, has attracted much interest among researchers. To date, numerous c-di-GMP effectors have been identified and well characterized. Generally, c-di-GMP effectors have emerged as riboswitches, PilZ domain-containing proteins, degenerate GGDEF and EAL domain- containing proteins, and transcriptional regulators from the perspective of structure and function [25]. Nevertheless, *in silico* analysis of Tsrp1 indicated it didn’t contain a conserved domain. Not only was Tsrp1 not a riboswitch, it also did not contain the PilZ domain. Neither the degenerate GGDEF nor the EAL domain was included in Tsrp1. In addition, we did not find a conserved DNA binding domain in Tsrp1. Thus, it was not recognized as a transcriptional regulator. Overall, Tsrp1 is a novel c-di-GMP effector protein in *R. ruber* SD3.

### Features of Recombinant *R. ruber* SD3 Enhancing the Expression of *tsrp1* Gene

**Fig. 3:**
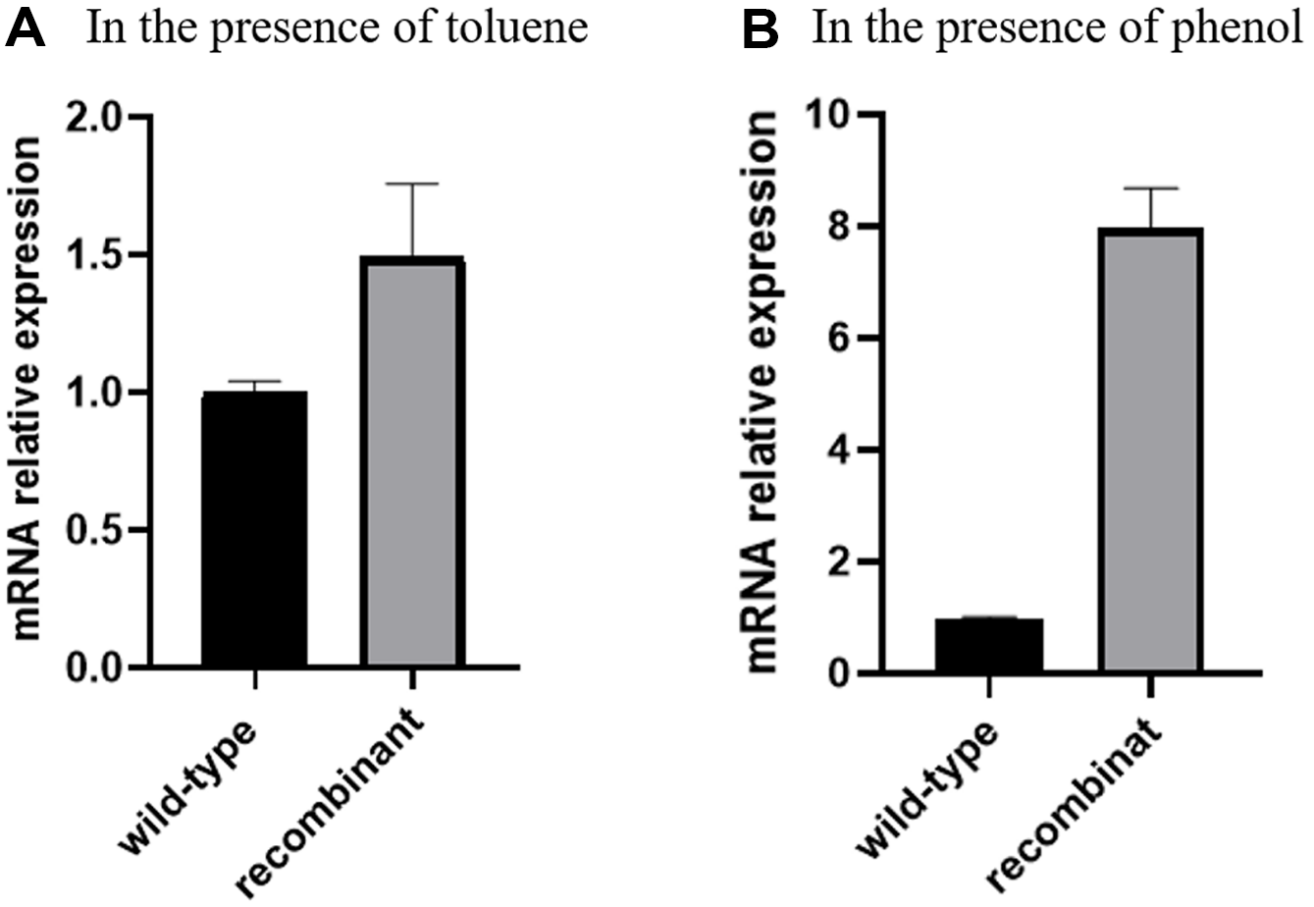
Change of mRNA relative expression of *tsrp1* gene in recombinant *R. ruber* SD3 and wild-type *R. ruber* SD3 in the presence of toluene (A) and phenol (B).

To reveal the influence of Tsrp1 on the organic solvent tolerance of *R. ruber* SD3, recombinant *R. ruber* SD3, which enhances the expression of *tsrp1* gene, was designed using a pNV18 shuttle plasmid. The shuttle plasmid was constructed by Chiba *et al*. and has several unique features for application in *Nocardia* spp. [26]. Afterwards, it was used to overproduce several proteins, such as GroEL-GroES [27], sigma 70 [28], and ribosomal protein L22 [22], in *R. ruber*. Herein, we also utilized a plasmid to increase the expression of Tsrp1. The results of quantitative real-time polymerase chain reaction demonstrated that the expression of the *tsrp1* gene in recombinant *R. ruber* SD3 was 1.49 and 7.97 times greater than that in wild-type *R. ruber* SD3 in the presence of toluene and phenol, respectively (Fig. 3). Generally, the process of gene expression in prokaryotes mainly includes two stages: transcription and translation. The gene transcription process requires the coordination and cooperation of components such as RNA polymerase, DNA template, and NTP substrates. When microorganisms are subjected to external stress, they may alter metabolic pathways within the cell, activate antioxidant systems, enhance DNA repair systems, and affect protein folding and degradation. It is likely these changes have an impact on the function or content of RNA polymerase, DNA template, and NTP substrates. Thus, the differences in Tsrp1 expression levels between the recombinant *R. ruber* SD3 and wild-type *R. ruber* SD3 strains under toluene and phenol exposure was observed via quantitative real-time polymerase chain reaction.

Next, we investigated whether the overproduction of Tsrp1 affected the morphology and growth of cells. Scanning electron microscope revealed no obvious difference in the morphology of the recombinant *R. ruber* SD3 and wild-type *R. ruber* SD3 strains (Fig. S2). Additionally, the growth of recombinant *R. ruber* SD3 was similar to that of wild-type *R. ruber* SD3 (data not shown).

**Fig. 4:**
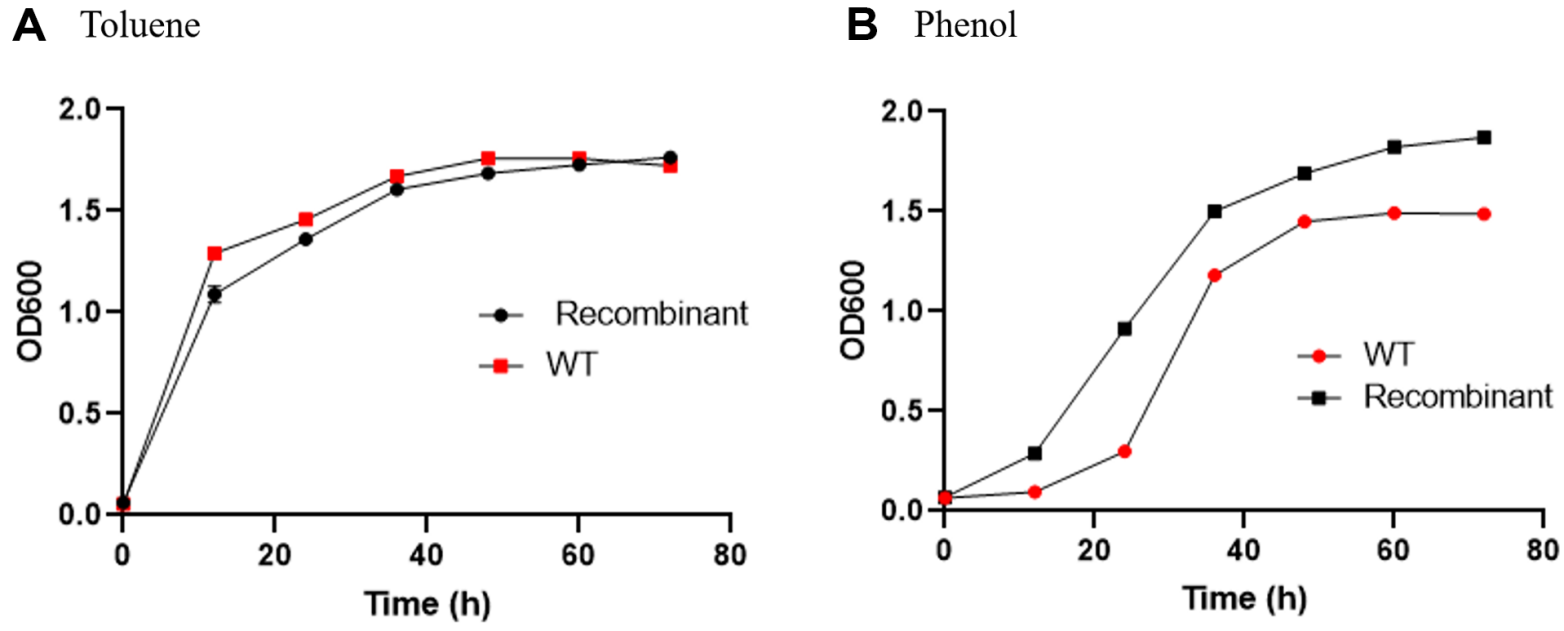
Growth of recombinant *R. ruber* SD3 and wild-type *R. ruber* SD3 in the presence of toluene and phenol. Toluene (0.02%, v/v) and phenol (0.08%, m/v) were separately added to LB broth. The cultivation was performed at 35°C and 200 r/min for 24 h and 48 h. Cultures were withdrawn to measure the OD at 600 nm. All assays were performed in duplicates. Experimental data were presented as mean ± SD.

**Fig. 5:**
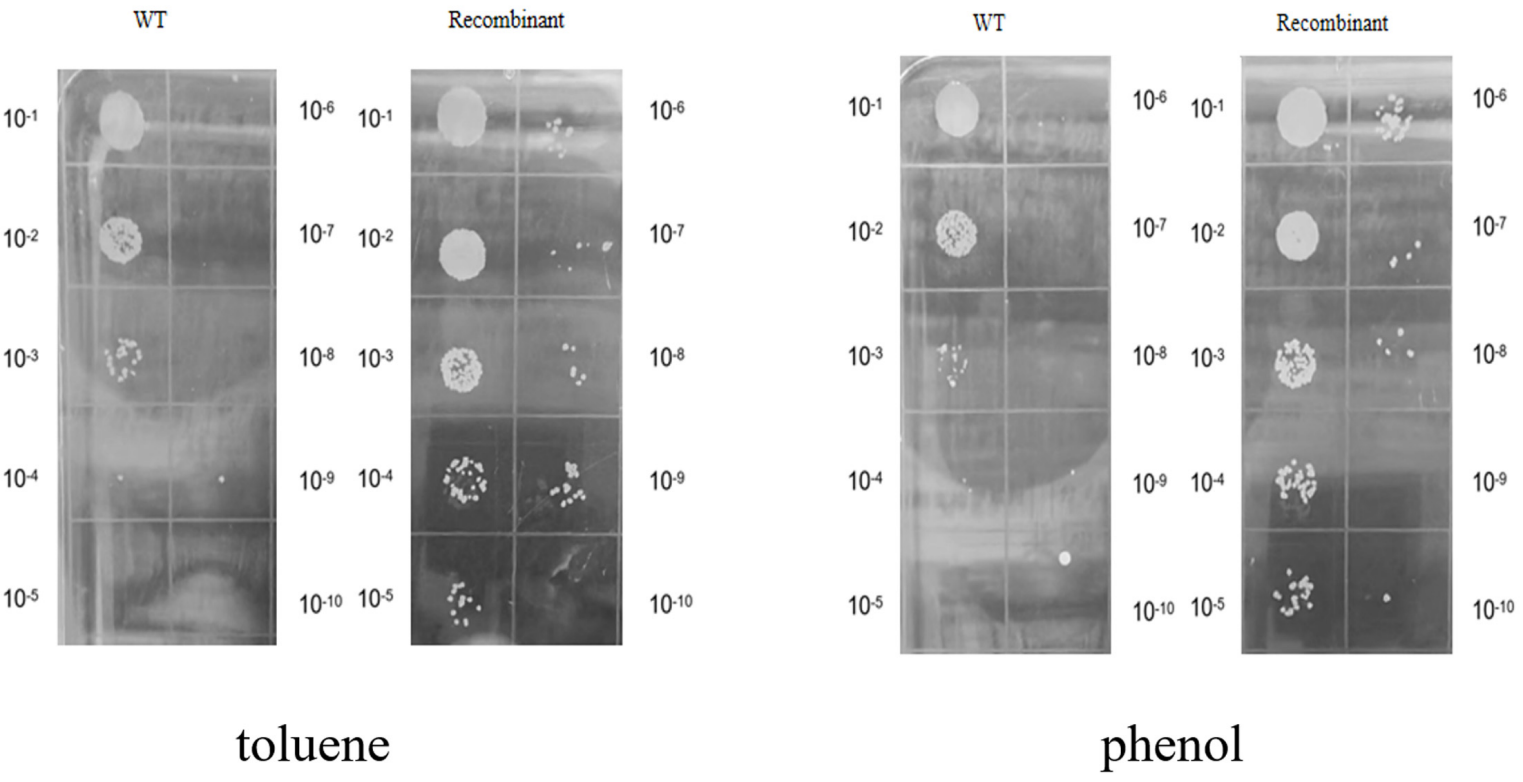
Colonies of recombinant *R. ruber* SD3 and wild-type *R. ruber* SD3 grown at 35°C for 12 h on agar plate in presence of toluene (300 μl, 12.5% v/v) and phenol (0.08% m/v), respectively.

Toluene and phenol are common organic solvents with industrial relevance [29]. The growth of recombinant *R. ruber* SD3 and wild-type *R. ruber* SD3 was compared in the presence of 0.02% (v/v) toluene and 0.08% (m/v) phenol, respectively. The results indicated that the growth of the recombinant *R. ruber* SD3 was similar to that of its counterpart under toluene stress (Fig. 4A), while the phenol tolerance of the recombinant *R. ruber* SD3 increased (Fig. 4B). We also investigated the effects of toluene and phenol on the growth of recombinant *R. ruber* SD3 and wild-type *R. ruber* SD3 on agar plates. A difference in colony number was observed between both strains in the presence of toluene and phenol (Fig. 5). There are more colonies of recombinant *R. ruber* SD3 than of the wild type under toluene and phenol stress. Nevertheless, both strains have similar abilities in the degradation of toluene and phenol. Both of recombinant *R. ruber* and the wild-type strain could completely degrade toluene (0.3 g/l, 0.6 g/l and 0.9 g/l) and phenol (0.6 g/l, 0.8 g/l and 1.0 g/l) in 72 h.

The above results suggested that it is feasible to enhance organic solvent tolerance by manipulating the expression of *tsrp1* gene in *R. ruber* SD3. Recombinant *R. ruber* had higher Tsrp1 expression levels compared with wild-type *R. ruber* when exposed to phenol or toluene. Different levels of Tsrp1 expression may affect the expression of other genes, thereby enabling the recombinant bacteria to better resist the toxicity of phenol or toluene. Hence it resulted in faster cell division and better growth of the recombinant *R. ruber*. Currently, some genes responsible for organic solvent tolerance and degradation have been identified in different microbes. These genes are associated with degradation and conversion of organic solvents via cellular metabolism [30], facilitating the folding of other proteins [31], discharging of organic solvents from cells [32], and decreasing cell membrane fluidity and cell surface hydrophobicity [33]. Interestingly, we discovered that Tsrp1 increased organic solvent tolerance and degradation in *R. ruber* SD3. As described above, Tsrp1 is a novel c-di-GMP effector protein. In some studies, c-di-GMP effectors were reported to participate in physiological functions, including development, morphogenesis, motile-sessile transition, biofilm formation, and bacterial virulence [28]. In *C. crescentus*, after TipF binds to c-di-GMP, it then binds TipN to localize to the cell pole. Following its recruitment of the flagellar components, flagellar assembly is initiated [34]. In *E. coli*, following binding to c-di-GMP, YcgR interacts with flagellar motors, thereby obstructing motor function [35]. In *C. difficile*, host colonization is regulated by c-di-GMP-mediated riboswitches [36]. Nevertheless, few data about the relationship between the c-di-GMP effector protein and organic solvent tolerance are available. Here, we revealed a link between organic solvent tolerance and a new c-di-GMP effector protein (Tsrp1) in *R. ruber* SD3.

### GO and KEGG Annotation Analysis of Differentially Expressed Genes (DEGs) between Recombinant *R. ruber* SD3 and Wild-Type *R. ruber* SD3

To explore how Tsrp1 in *R. ruber* SD3 affects organic solvent tolerance, RNA-seq of recombinant *R. ruber* SD3 and wild-type *R. ruber* SD3 under 0.08% (m/v) phenol stress was performed. All unigenes were annotated using six databases (NCBI nonredundant protein/nucleotide sequences, Protein family, Clusters of Orthologous Groups of proteins, Swiss-Prot, Kyoto Encyclopedia of Genes and Genomes, Gene Ontology). A total of 4,827 unigenes were successfully annotated. According to the definition of differentially expressed genes, 869 genes were identified as DEGs between recombinant *R. ruber* SD3 and wild-type *R. ruber* SD3. Among the 869 DEGs, 454 genes were upregulated, while 415 genes were downregulated.

The Gene Ontology is a community-based platform that supplies data about the function of the gene products [37]. For the top 20 GO terms of DEGs, two GO terms were associated with biological processes, three GO terms were associated with cellular components and fifteen GO terms belonged to molecular functions (Fig. S3). According to the cellular component, 137 DEGs were categorized into the integral component of membrane, accounting for the largest portion of the cellular component. In terms of molecular function, 74 DEGs were mostly associated with DNA binding (Fig. S3). Generally, organic solvents have a profound impact on the cell membrane and the bacterial chromosome organization and segregation. Membrane proteins associated with transporter, signal transduction, as well as energy and material metabolism, play a role in the adaptation to organic solvent tolerance [38]. Moreover, DNA binding proteins often have specific domains that facilitate their interaction with DNA, and their function is crucial for maintaining the integrity and regulation of the genetic material within cells. Hence, it is not surprising that the DEGs were mostly categorized as integral components of membrane and DNA binding.

KEGG is a resource for the systematic analysis of gene functions, in which genomic data are linked with functional data within a higher order [39]. According to the KEGG database, 253 DEGs were linked to metabolism, 30 were related to genetic information processing, 34 were associated with environmental information processing, 12 were related to cellular processes, 15 were involved in organismal systems, and 10 were connected with human diseases (Fig. S4). Most DEGs were annotated to metabolism. Interestingly, 39 DEGs were closely associated with xenobiotic biodegradation and metabolism. The processes included the degradation of the following: benzoate (22 DEGs), chlorocyclohexane and chlorobenzene (9 DEGs), xylene (6 DEGs), fluorobenzoate (6 DEGs), styrene (5 DEGs), steroids (4 DEGs), chloroalkane and chloroalkene (3 DEGs), and toluene (3 DEGs). These results indicated that Tsrp1 may markedly influence the organic solvent tolerance and degradation of *R. ruber* SD3.

### GO and KEGG Enrichment Analysis of DEGs between Recombinant *R. ruber* SD3 and Wild-Type *R. ruber* SD3

To classify the functions of the DEGs, GO and KEGG enrichment analyses were conducted. The top 20 significantly enriched GO terms are shown in Fig. S5. Six enriched GO terms were associated with biological processes, 10 pertained to molecular function, and 4 were connected to cellular components. Among them, the significantly enriched GO terms were associated with oxidoreductase and protein synthesis. This suggested that organic solvent stress affected cell redox homeostasis and intracellular protein metabolism.

Fig. S6 illustrated the KEGG enrichment analysis of differentially expressed genes between the recombinant *R. ruber* SD3 and the wild type. It was noteworthy that within the metabolic pathways, 194 upregulated genes and 194 downregulated genes were enriched. There were 12, 6, 3, 3, and 2 upregulated genes that were enriched in degrading benzoate, chlorocyclohexane and chlorobenzene, toluene, fluorobenzoate, and dioxin. This suggests that Tsrp1 influences the metabolism of numerous pollutants. Additionally, there was an enrichment of upregulated genes associated with ABC transporters and the ribosome, indicating that Tsrp1 likely plays a role in altering material transport within cells and in the process of protein translation.

### Differential Expression of Key Genes Involved in Toluene Degradation and Transcription Factors between Recombinant *R. ruber* SD3 and Wild-Type *R. ruber* SD3

**Fig. 6:**
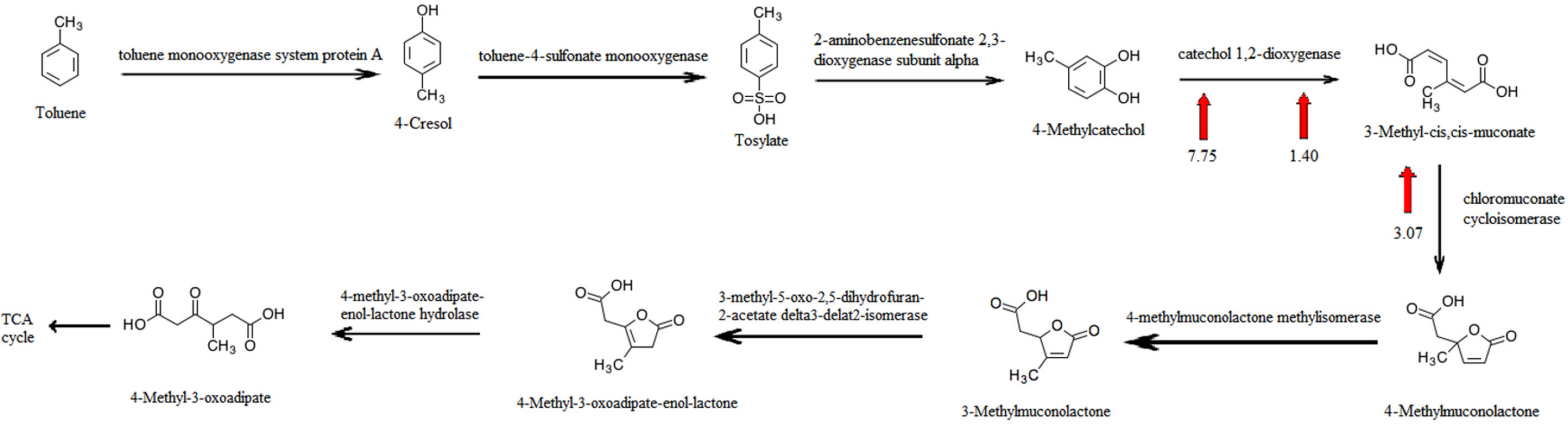
Pathway illustrating general mechanisms of toluene degradation in *R. ruber* and the observed expression level changes between recombinant *R. ruber* SD3 and wild-type *R. ruber* SD3.

Toluene degradation was carried out in two different ways. In some bacteria, the action of a dioxygenase first results in the production of a cis-dihydrodiol, and then dehydrogenation is catalyzed to form 3-methylcatechol. Meanwhile, in other bacteria the hydroxylation at the ortho, meta, or para position of toluene is initiated by monooxygenases, and then a second hydroxylation by the same or a different hydroxylase is subjected to the formation of catecholic products [40]. Catechol dioxygenases are iron-containing oxidases that can open the structure of the benzene ring and add oxygen atoms to the carbon atoms by breaking bonds, thereby converting aromatic hydrocarbon carbon into long chains and eventually degrading it into carbon dioxide and water. Catechol dioxygenases are divided into catechol 1,2-dioxygenases and catechol 2, 3-dioxygenases. Catechol 1,2-dioxygenase is the key enzyme of the ortho-position cleavage pathway, which results in the formation of cis, cis-muconic acid. Chloromuconate cycloisomerase also displayed activity toward muconate cycloisomerase, which can transform cis, cis- muconic acid into (S)-muconolactone. Subsequently, the metabolites formed from toluene entered the tricarboxylic acid pathway [41]. Moreover, similar to *Frateuria* species ANA-18 [42] and *Acinetobacter lwoffii* K24 [43], *R. ruber* SD3 contains two different kinds of catechol 1,2-dioxygenases, which suggests that this enzyme plays an important role in toluene degradation (Fig. 6). According to the transcriptome results, three DEGs related to toluene degradation, namely two kinds of catechol 1,2-dioxygenase and a chloromuconate cycloisomerase, were upregulated in recombinant *R. ruber* SD3. Compared with those of wild-type *R. ruber* SD3, the Log2FC values of these proteins were 7.75, 1.40, and 3.07. Furthermore, 2-oxo acid dehydrogenase subunit E2, α-ketoacid dehydrogenase subunit β, and pyruvate dehydrogenase (acetyl-transferring) E1 component subunit α involved in TCA cycle also had higher expression in recombinant *R. ruber* SD3 compared with the wild type. The enhanced expression of these proteins illustrated increased toluene degradation in recombinant *R. ruber* SD3 (Fig. 6).

TFs refer to the proteins that bind to the specific nucleotide sequences upstream of genes for the regulation of gene transcription. P2TF is an integrated and comprehensive database of TF proteins. This tool supplies detailed information on each TF gene, such as its classification, sequence features, functional domains, and chromosomal localization. Thus, it is used for the prediction of prokaryotic transcription factors [44]. Here, we also used P2TF to predict transcription factors among the DEGs. As indicated in Tables S2 and S3, 49 upregulated and 42 downregulated DEGs in recombinant *R. ruber* SD3 were predicted to be TFs. Among 49 upregulated TFs, 35 TFs, seven TFs, four TFs, two TFs and one TF were included in the TR class, OCS class, SF class, nan class, and ODP class, respectively. Based on the gene type, these TFs mainly belonged to the following families: TetR, WhiB, MarR, LysR, AraC, and GntR. Forty-two downregulated TFs were included in the TetR, MarR, PadR, DeoR, AraC, IclR, and GntR families. It was reported that transcriptional regulators in the TetR, LysR, AraC, IclR, and GntR families were associated with the degradation of aromatic compounds [13, 45]. The results in Tables S2 and S3 indicated that many TFs that regulated aromatic catabolic pathways were differentially expressed in recombinant *R. ruber* SD3. Among all the differentially expressed TFs, 17 belonged to the TetR family, whose transcriptional regulators are well known for their ability to control genes encoding small-molecule exporters [46]. Effluxing is regarded as an efficient way to increase solvent tolerance. In some bacteria, efflux pumps play crucial roles in solvent tolerance [32]. For instance, *P. putida* was not completely grown in the presence of toluene owing to the knockout of genes encoding the RND-type efflux pump TtgGHI [47]. In recombinant *R. ruber* SD3, the differential expression of the TetR family transcriptional regulators may indicate that these proteins were associated with the regulation of the efflux pump.

Furthermore, TetR family transcriptional regulators are also related to fatty acid metabolism and the metabolism of other lipids, such as sterols [46]. Microbial organic solvent tolerance is modulated by a shift in the saturated/unsaturated fatty acid ratio, cis-trans isomerization of unsaturated fatty acids, and a variation in phospholipid polarity heads in the cell membrane [48]. Thus, TetR family transcriptional regulators are differentially expressed and can change fatty acid and/or other lipid metabolism, thereby altering the components of the cell membrane to adapt to organic solvent stress.

### RT–qPCR Verification of DEGs Between Recombinant *R. ruber* SD3 and Wild-Type *R. ruber* SD3

To demonstrate the transcriptome results described above, the transcription levels of several TF genes in recombinant *R. ruber* SD3 and wild-type *R. ruber* SD3 were assayed by using RT–qPCR (Table S4). Comparison of the expression variation of some DEGs assayed by transcriptome sequencing and RT–qPCR indicated that the transcriptional changes measured using two different methods were related to each other to some extent, with a Pearson correlation coefficient of 0.3752. However, the data obtained using RNA-Seq and qPCR were not entirely consistent. One reason was that RNA-Seq and qPCR differed in their experimental procedures. Another reason was that gene expression patterns exhibited complexity and diversity, with some genes showing differential changes in multiple transcripts during RNA-Seq analysis. In addition, some genes with low expression levels gave rise to differences in the results.

Whole-transcriptome analysis plays an essential role in revealing the functions of proteins that respond to environmental challenges. Using RNA-Seq, Song *et al*. reported the role of IrrE in the organic solvent tolerance of *Arthrobacter* simplex [49]. Transcriptome analysis revealed DEGs under 1-butanol treatment [50]. Here, we used a transcriptome method to verify that Tsrp1 affected the expression of many genes. Our findings suggested that Tsrp1 may influence the expression of transcription factors, efflux pumps, and some proteins related to xenobiotic biodegradation and cell membrane components, thereby regulating the tolerance of *R. ruber* SD3 to organic solvents.

## Supplemental Materials

Supplementary data for this paper are available on-line only at http://jmb.or.kr.
