# Supplementary material for: Tsrp1 Is a Novel Cyclic Diguanosine Monophosphate Effector that Plays a Role in the Organic-Solvent Tolerance of Rhodococcus ruber
Source: J Microbiol Biotechnol. 2025 Mar 26;35:e2410039. doi: 10.4014/jmb.2410.10039 (PMC11985408; doi:10.4014/jmb.2410.10039)
Supplement: Supplementary file 1 [file jmb-35-e2410039-supple.pdf]

J. Microbiol. Biotechnol. 2025. 35: e2410039
https://doi.org/10.4014/jmb.2410.10039

Supplementary Tables and Figures

Table S1. Sequences of the primers used in the study.

Gene_id	Gene description	**Forward sequence (5'-3')**	**Reverse sequence (5'-3')**
DCN13_22360	IclR family transcriptional regulator	ACGGTTCCTGCTCACCCTGG	ACGTCGACTCGTGCACCTTCTG
DCN13_24285	LuxR family transcriptional regulator	ACGAGCAGCCGCCACTA	CAACCCTTCCCCGATGT
DCN13_16390	TetR family transcriptional regulator	GTTCCGCAGCAGCGCACAT	CGAACGCCGCAGCCACA
DCN13_10115	metal-dependent transcriptional regulator	GGGAATGGTCGGGTGAGAAGG	CGCCGTAGCGTGCGTGGTC
DCN13_11115	MerR family DNA-binding transcriptional regulator	CCGAGGTTCTGGGCATACTGGC	CAAGCGTGCGGCTGCGTC
DCN13_15250	WhiB family transcriptional regulator	CGCGTGCGCGAGTTGCC	CCCCACACACCCCACGGCT
DCN13_17830	XRE family transcriptional regulator	GACGGAGGGGACGATGACAAG	GCTGCGAGCGGATGAAACTG
DCN13_22320	MarR family transcriptional regulator	GCTGCCCCTGCTCCTGTTCG	GCCTGCTTGGAGACGCCGA
DCN13_12115	XRE family transcriptional regulator	CGACCTGGTTCTCTCCCCCG	TCGTTCACCCTGCTTGCCG
DCN13_02005	GntR family transcriptional regulator	AGGTCTCGTGGAGTCGG	GCAGCCAGAAGATGTCG
	16SrDNA	ACTGGGCGTAAAGAGYTCGT	CGCATTTCACCGCTACAC
	Tsrp1	CCGCCGAGTTCACCAAGG	GGTATCGACGGTCAGGTTGC

Table S2. The upregulated DEGs in recombinant *R. ruber* SD3 predicted to be TFs with P2TF.

Gene ID	Gene description	P2TF description	$\log_2$FC
DCN13_00235	TetR family transcriptional regulator	Transcription factor, TetR family contains 1 TetR_N	1.63
DCN13_04630	hypothetical protein	Transcription factor, WhiB family contains 1 Whib	1.57
DCN13_22505	AraC family transcriptional regulator	Transcription factor, AraC family contains 2 HTH_AraC	1.52
DCN13_20420	diguanylate cyclase	Transcription factor, Unclassified contains 1 GAF,1 Trans_reg_C	1.15
DCN13_10105	transposase	Sigma factor, Unclassified contains 1 Sigma70_r4	1.28
DCN13_24410	helix-turn-helix domain-containing protein	DNA-binding protein, Unclassified contains 1 MerR,1 TOBE	3.91
DCN13_18440	AraC family transcriptional regulator	Transcription factor, AraC family contains 2 HTH_AraC	2.32
DCN13_09735	LysR family transcriptional regulator	Transcription factor, LysR family contains 1 HTH_1,1 LysR_substrate	1.66
DCN13_21015	PLP-dependent aminotransferase family protein	Transcription factor, GntR family contains 1 GntR,1 Aminotran_1_2	1.45
DCN13_15075	WhiB family transcriptional regulator	Transcription factor, WhiB family contains 1 Whib	1.11
DCN13_18015	SigB/SigF/SigG family RNA polymerase sigma factor	Sigma factor, RpoD family contains 1 Sigma70_r2,1 Sigma70_r3,1 Sigma70_r4	1.29
DCN13_23150	TetR/AcrR family transcriptional regulator	Transcription factor, TetR family contains 1 TetR_N	1.36
DCN13_15250	WhiB family transcriptional regulator	Transcription factor, WhiB family contains 1 Whib	2.26
DCN13_09465	WhiB family transcriptional regulator	Transcription factor, WhiB family contains 1 Whib	1.34
DCN13_10115	metal-dependent transcriptional regulator	DNA-binding protein, Unclassified contains 1 HTH_DTXR,1 FeoA	1.72
DCN13_13640	TetR/AcrR family transcriptional regulator	Transcription factor, TetR family contains 1 TetR_N	1.42
DCN13_11510	carbamoyl-phosphate synthase small subunit	DNA-binding protein, Bhl family contains 1 Bac_DNA_binding	1.29
DCN13_11515	carbamoyl-phosphate synthase large subunit	Transcription factor, LuxR family contains 1 HTH_LUXR	1.46
DCN13_17810	alpha/beta hydrolase	Transcription factor, GutM family contains 1 GutM	1.26
DCN13_02005	GntR family transcriptional regulator	Transcription factor, GntR family contains 1 GntR	1.23
DCN13_16390	TetR family transcriptional regulator	Transcription factor, TetR family contains 1 TetR_N	2.04
DCN13_07575	IclR family transcriptional regulator	Transcription factor, IclR family contains 1 HTH_IclR,1 IclR	1.49
DCN13_16245	RNA polymerase sigma factor ShbA	Sigma factor, Ecf family contains 1 Sigma70_r2,1 Sigma70_r4	1.48
DCN13_00635	TetR/AcrR family transcriptional regulator	Transcription factor, TetR family contains 1 TetR_N	1.46
DCN13_12115	XRE family transcriptional regulator	Transcription factor, Xre family contains 1 HTH_XRE	1.19
DCN13_22330	CoA transferase subunit B	Transcription factor, DeoR family contains 1 DeoR	4.1
DCN13_11115	MerR family DNA-binding transcriptional regulator	Transcription factor, MerR family contains 1 MerR,1 MerR-DNA-bind	1.89
DCN13_07475	LysR family transcriptional regulator	Transcription factor, LysR family contains 1 HTH_1,1 LysR_substrate	1.29
DCN13_02375	catalase	Transcription factor, Fur family contains 1 FUR	1.59
DCN13_02370	transcriptional repressor	Transcription factor, Fur family contains 1 FUR	1.90
DCN13_21915	methylated-DNA--[protein]-cysteine	Transcription factor, AraC family contains 1 HTH_AraC	1.19

	S-methyltransferase		
DCN13_03200	cell division protein ZapE	Transcription factor, Unclassified contains 1 Bac_DnaA	1．60
DCN13_15940	peptidoglycan endopeptidase	Transcription factor, AraC family contains 1 AraC_N	1．12
DCN13_04055	DNA-binding transcriptional regulator OxyR	Transcription factor, LysR family contains 1 HTH_1,1 LysR_substrate	2．21
DCN13_22360	IclR family transcriptional regulator	Transcription factor, IclR family contains 1 HTH_IclR,1 IclR	2．49
DCN13_11650	triose-phosphate isomerase	Transcription factor, Xre family contains 1 HTH_XRE	1．29
DCN13_01655	LysR family transcriptional regulator	Transcription factor, LysR family contains 1 HTH_1,1 LysR_substrate	1．30
DCN13_13625	TetR/AcrR family transcriptional regulator	Transcription factor, TetR family contains 1 TetR_N	1．37
DCN13_24285	LuxR family transcriptional regulator	Transcription factor, LuxR family contains 1 HTH_LUXR	1．85
DCN13_24450	RNA polymerase sigma factor SigM	Sigma factor, Ecf family contains 1 Sigma70_r2,1 Sigma70_r4	1．07
DCN13_17830	XRE family transcriptional regulator	Transcription factor, Xre family contains 1 HTH_XRE	1．12
DCN13_19680	LuxR family transcriptional regulator	Transcription factor, LuxR family contains 1 HTH_LUXR	1．31
DCN13_22320	MarR family transcriptional regulator	Transcription factor, MarR family contains 1 MarR	1．71
DCN13_13335	MarR family transcriptional regulator	Transcription factor, MarR family contains 1 MarR	1．11
DCN13_14905	ArsR family transcriptional regulator	Transcription factor, ArsR family contains 1 HTH_5	1．07
DCN13_14900	VOC family protein	Transcription factor, ArsR family contains 1 HTH_5,1 Glyoxalase	1．12
DCN13_03900	MarR family transcriptional regulator	Transcription factor, MarR family contains 1 MarR	1．20
DCN13_03995	ArsR family transcriptional regulator	Transcription factor, ArsR family contains 1 HTH_5	1．64
DCN13_00640	ATP-grasp domain-containing protein	Transcription factor, PucR family contains 1 PucR	1．31

Table S3. The downregulated DEGs in recombinant *R. ruber* SD3 predicted to be TFs with P2TF.

Gene ID	Gene description	P2TF description	log$_2$FC
DCN13_09370	TetR/AcrR family transcriptional regulator	Transcription factor, TetR family contains 1 TetR_N	−1.02
DCN13_19240	PadR family transcriptional regulator	Transcription factor, PadR family contains 1 PadR	−1.52
DCN13_16255	WhiB family transcriptional regulator	Transcription factor, WhiB family contains 1 Whib	−1.46
DCN13_13250	MerR family DNA-binding transcriptional regulator	Transcription factor, MerR family contains 1 MerR	−1.68
DCN13_05515	TetR/AcrR family transcriptional regulator	Transcription factor, TetR family contains 1 TetR_N	−1.23
DCN13_21165	RNA polymerase subunit sigma-70	Sigma factor, Ecf family contains 1 Sigma70_r2,1 Sigma70_r4	−1.22
DCN13_17655	AraC family transcriptional regulator	Transcription factor, AraC family contains 1 HTH_AraC	−2.72
DCN13_10155	helix-turn-helix transcriptional regulator	Transcription factor, LuxR family contains 1 HTH_LUXR	−1.03
DCN13_06990	PucR family transcriptional regulator	DNA-binding protein, Fis family contains 1 HTH_8	−1.28
DCN13_22225	TetR/AcrR family transcriptional regulator	Transcription factor, TetR family contains 1 TetR_N	−1.64
DCN13_01535	TetR/AcrR family transcriptional regulator	Transcription factor, TetR family contains 1 TetR_N	−1.11
DCN13_02340	TetR/AcrR family transcriptional regulator	Transcription factor, TetR family contains 1 TetR_N	−3.16
DCN13_13215	acetate--CoA ligase	Transcription factor, RpiR family contains 1 HTH_6	−1.52
DCN13_08980	PadR family transcriptional regulator	Transcription factor, PadR family contains 1 PadR	−2.49
DCN13_19040	pyridoxal phosphate-dependent aminotransferase	Transcription factor, Xre family contains 1 HTH_XRE,1 Aminotran_1_2	−1.08
DCN13_10440	DeoR/GlpR transcriptional regulator	Transcription factor, DeoR family contains 1 HTH_DeoR,1 DeoR	−1.28
DCN13_08230	MarR family transcriptional regulator	Transcription factor, MarR family contains 1 MarR	−2.01
DCN13_01405	MarR family transcriptional regulator	Transcription factor, MarR family contains 1 MarR	−1.21
DCN13_24370	TetR/AcrR family transcriptional regulator	Transcription factor, TetR family contains 1 TetR_N	−1.04
DCN13_15760	Lrp/AsnC family transcriptional regulator	Transcription factor, MarR family contains 1 MarR,1 AsnC_trans_reg	−1.40
DCN13_17430	helix-turn-helix domain-containing protein	DNA-binding protein, Unclassified contains 1 MerR	−1.24
DCN13_20745	TetR/AcrR family transcriptional regulator	Transcription factor, TetR family contains 1 TetR_N	−3.11
DCN13_10695	MarR family transcriptional regulator	Transcription factor, MarR family contains 1 MarR	−1.07
DCN13_20260	TetR/AcrR family transcriptional regulator	Transcription factor, TetR family contains 1 TetR_N	−1.38
DCN13_24065	PucR family transcriptional regulator	Transcription factor, PucR family contains 1 PucR	−1.19
DCN13_17410	helix-turn-helix domain-containing protein	Transcription factor, Unclassified contains 1 HTH_11	−1.41
DCN13_14435	TetR family transcriptional regulator	Transcription factor, TetR family contains 1 TetR_N	−1.47
DCN13_12400	non-ribosomal peptide synthetase	Transcription factor, AsnC family contains 1 AsnC_trans_reg	−1.52

DCN13_23465	TetR family transcriptional regulator	Transcription factor, TetR family contains 1 TetR_N	−1.47
DCN13_14395	ArsR family transcriptional regulator	Transcription factor, ArsR family contains 1 HTH_5	−1.53
DCN13_12995	excinuclease ABC subunit B	DNA-binding protein, Hns family contains 1 Histone_HNS	−1.05
DCN13_00900	enoyl-CoA hydratase	Transcription factor, GntR family contains 1 GntR	−1.41
DCN13_01935	acetyl-CoA C-acetyltransferase	Transcription factor, Unclassified contains 1 BetR	−1.08
DCN13_16730	ethanolamine permease	Transcription factor, PadR family contains 1 PadR	−2.18
DCN13_13140	response regulator	Response regulator, NarL family contains 1 Response_reg,1 HTH_LUXR	−1.07
DCN13_24105	glutamate dehydrogenase	Transcription factor, DeoR family contains 1 DeoR	−1.37
DCN13_08115	hypothetical protein	Transcription factor, Unclassified contains 1 Trans_reg_C	−1.05
DCN13_19485	MarR family transcriptional regulator	Transcription factor, MarR family contains 1 MarR	−1.17
DCN13_11490	bifunctional pyr operon transcriptional regulator/uracil phosphoribosyltransferase PyrR	DNA-binding protein, Unclassified contains 1 HTH_LUXR	−1.43
DCN13_07230	TetR/AcrR family transcriptional regulator	Transcription factor, TetR family contains 1 TetR_N	−3.28
DCN13_16160	phosphoribosyl-ATP pyrophosphohydrolase	DNA-binding protein, Hns family contains 1 Histone_HNS	−1.88
DCN13_01865	IclR family transcriptional regulator	Transcription factor, IclR family contains 1 HTH_IclR,1 IclR	−2.22

Table S4. Comparison of the transcription changes of some DEGs based on transcriptome sequencing and RT-qPCR.

Gene_id	Gene description	Transcriptome sequencing (log_2FC)	RT-qPCR (log_2FC)
DCN13_22360	IclR family transcriptional regulator	2.49	0.84
DCN13_24285	LuxR family transcriptional regulator	1.85	1.45
DCN13_16390	TetR family transcriptional regulator	2.04	1.46
DCN13_10115	metal-dependent transcriptional regulator	1.72	0.62
DCN13_15250	WhiB family transcriptional regulator	2.26	2.10
DCN13_17830	XRE family transcriptional regulator	1.12	1.18
DCN13_22320	MarR family transcriptional regulator	1.71	0.46
DCN13_12115	XRE family transcriptional regulator	1.19	0.57
DCN13_02005	GntR family transcriptional regulator	1.23	1.04

MTTAKTPVDAAVAKTTADAAKANEAAAKATADATAAAQKIAESATAAANQATAEFTKAL
DDSAARLSEFNDKVVDAAKQSGNLTVDTYERAVSSLLDLQEKLASASPVGWVEDLTKA
QVSFARELTATVTTTARDLLK

Fig. S1. Mass spectrometry identification of Tsrp1. Different colors represent different confidence levels of the sequence. The sequences shown in green, yellow, red and gray have confidence levels of more than 95%, 50~95%, 0~50% and zero, respectively.

Fig. S2. The morphology of recombinant *R. ruber* SD3 and wild-type *R. ruber* SD3 under a scanning electron microscope. (A) Recombinant *R. ruber* SD3; (B) wild-type *R. ruber* SD3.

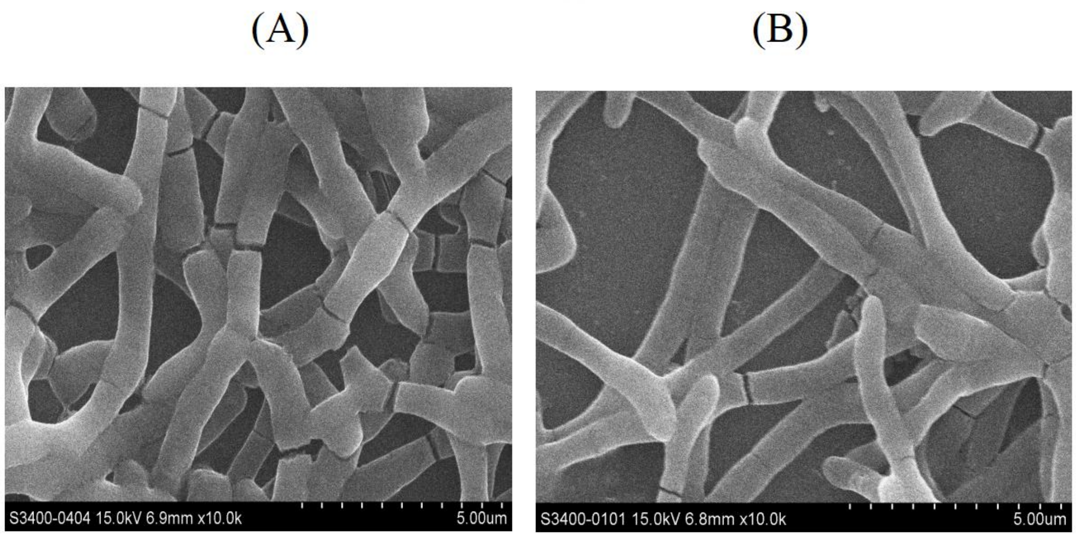

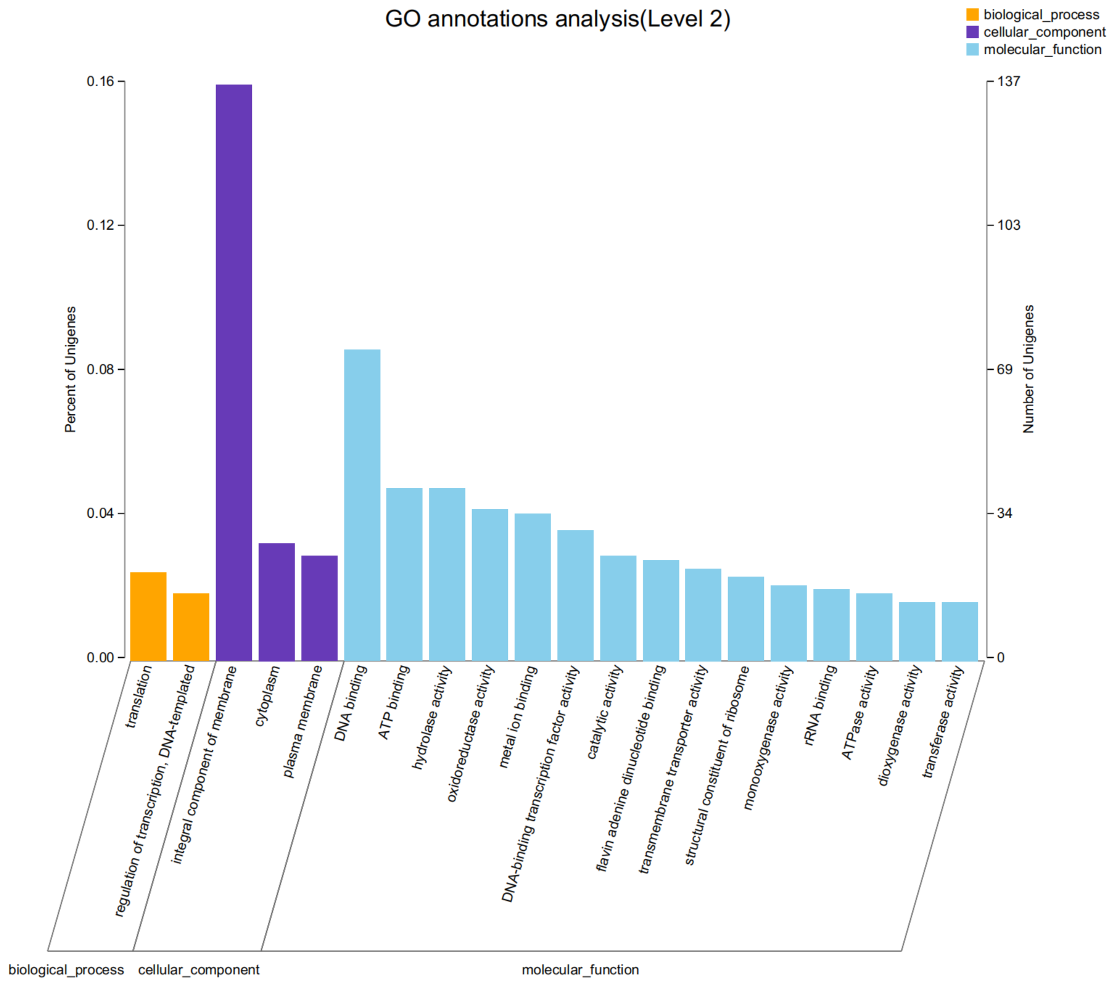

Fig. S3. GO annotation analysis of differentially expressed genes. The horizontal axis in the figure represents the secondary classification terms of GO. The left vertical axis represents the percentage of genes in the secondary classification, and the right vertical axis represents the number of genes aligned with the secondary classification. The three colors represent the three major categories.

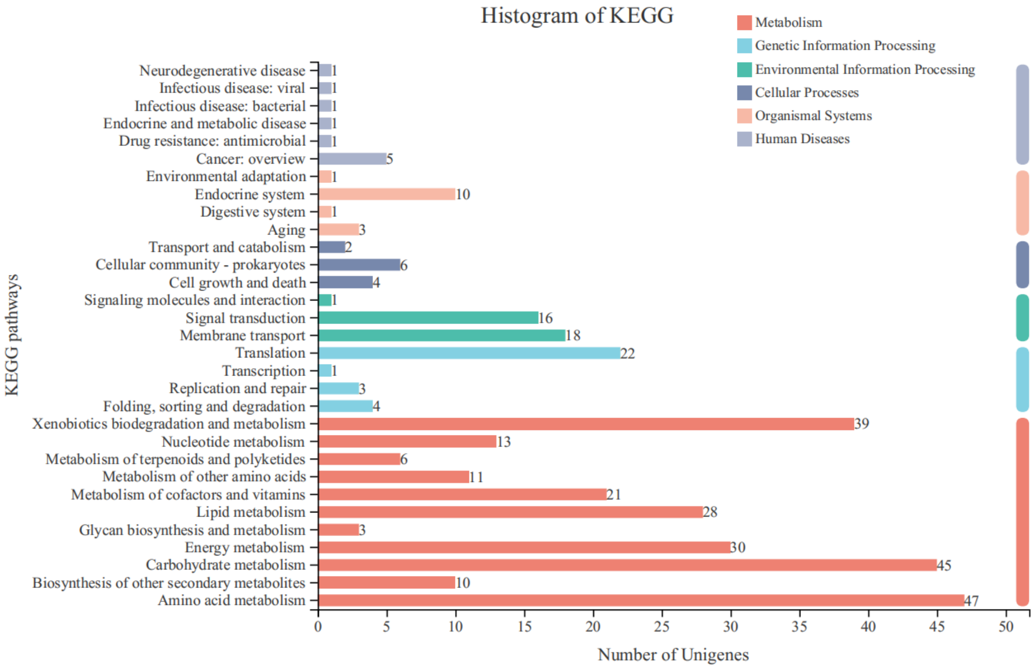

Fig. S4. KEGG annotation analysis of differentially expressed genes. The horizontal axis in the figure represents the name of the KEGG metabolic pathway, and the vertical axis in the figure represents the number of genes annotated in the pathway.

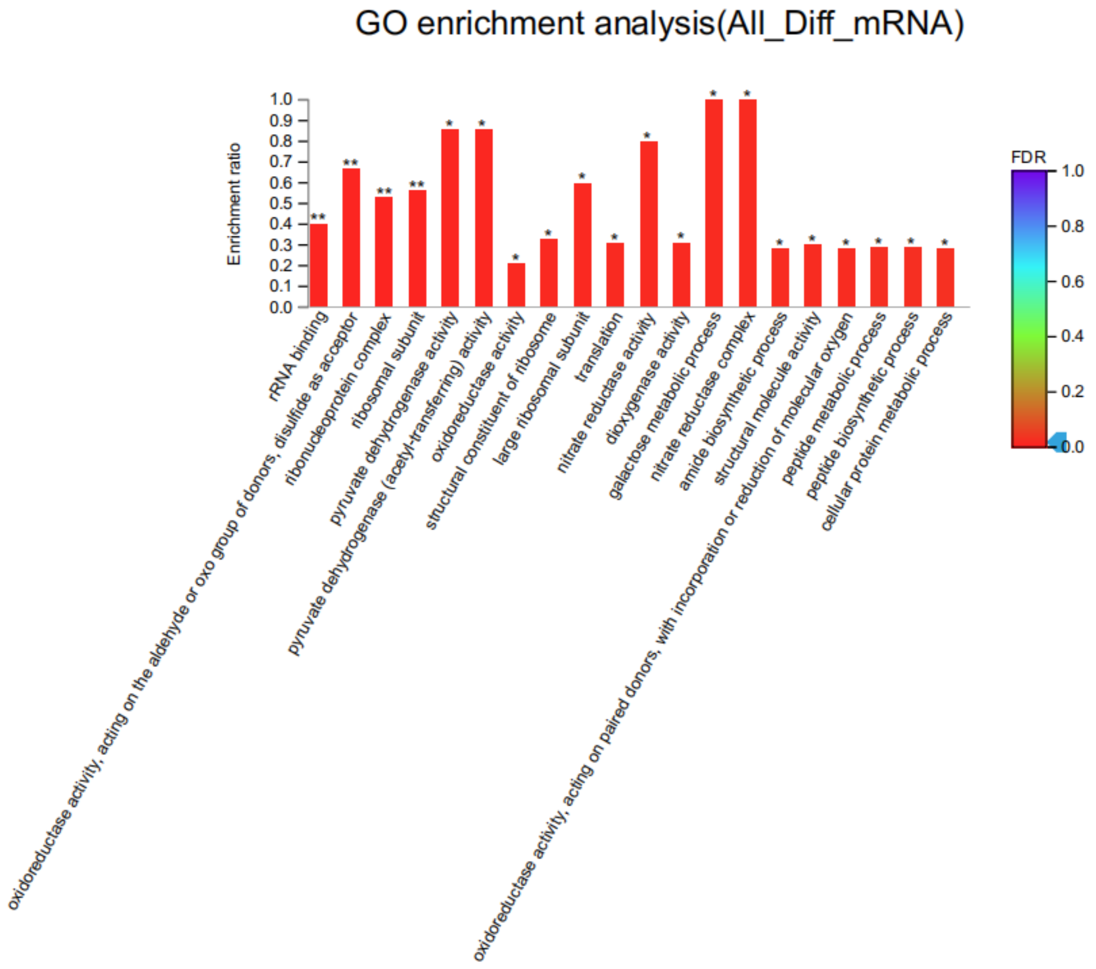

Fig. S5. GO enrichment analysis of differentially expressed genes. The horizontal axis in the figure represents GO terms. The vertical axis represents enrichment ratio. The color indicates the significance of the enrichment, where FDR < 0.001, FDR < 0.01 and FDR < 0.05 are marked ***, ** and *, respectively.

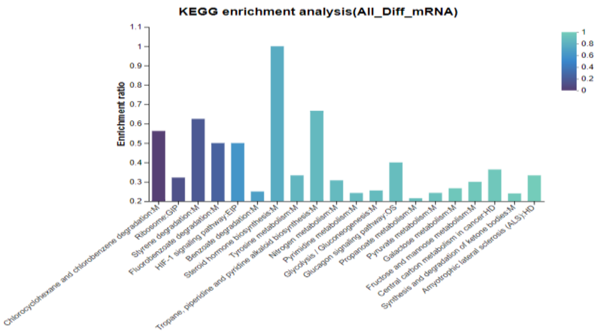

(A)

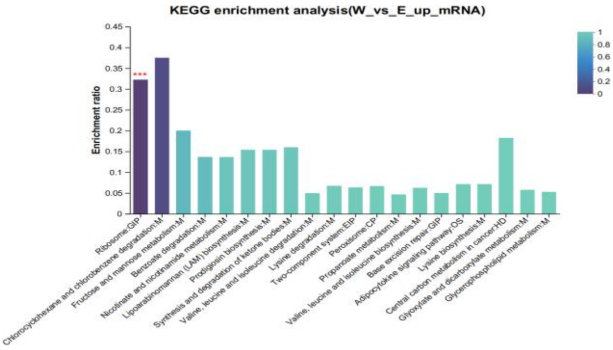

(B)

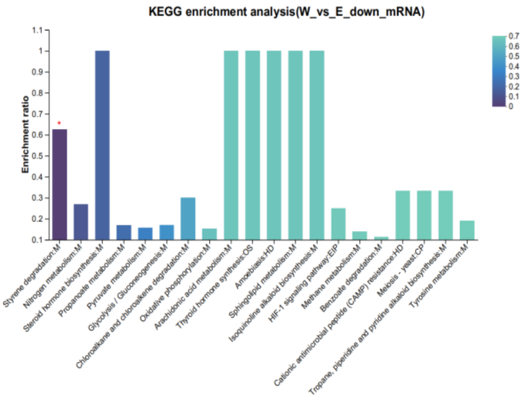

(C)

Fig. S6. KEGG enrichment analysis of differentially expressed genes. **(A)** The differentially expressed genes; **(B)** The upregulated differentially expressed genes; (**C)** the downregulated differentially expressed genes. The horizontal axis in the figure represents the names of pathways. The vertical axis represents enrichment ratio.
